# Bronchial mucosal nuclear transcription factor expression and inflammatory response in humans after exposure to wood smoke

**DOI:** 10.1186/s12989-026-00685-6

**Published:** 2026-06-12

**Authors:** Alva Hansson, Maria Friberg, Gregory Rankin, Jamshid Pourazar, Oskari Uski, Natxo García-López, Christoffer Boman, Anders Blomberg, Annelie Behndig, Thomas Sandström, Ala Muala

**Affiliations:** 1 Department of Public Health and Clinical Medicine, Umeå University, Umeå, Sweden; 2 Thermochemical Energy Conversion Laboratory, Department of Applied Physics and Electronics, Umeå University, Umeå, Sweden

**Keywords:** (3–10 st) Air pollution, Biomass combustion, Wood smoke, Controlled human exposure, Bronchoscopy, Endobronchial mucosal biopsies

## Abstract

**Background:**

Exposure to wood smoke is associated with negative respiratory health outcomes such as airway infections and development of chronic obstructive pulmonary disease (COPD). Previous controlled exposure studies in humans with bronchoscopy sampling have shown wood smoke-induced bronchial cytotoxicity and impaired macrophage phagocytosis. The present study investigated whether an early and transient acute inflammatory response, as reflected in bronchial mucosal biopsies and lavage fluids, could be detected 6 h after wood smoke exposure.

**Methods:**

On two separate occasions, fourteen healthy participants were exposed, in a double-blind, randomised crossover design, for 2 h to filtered air and diluted wood smoke generated from incomplete wood log combustion with a mean particulate matter concentration of 409 ± 43 µg/m^3^. Bronchoscopy with endobronchial mucosal biopsies, bronchial wash (BW) and bronchoalveolar lavage (BAL) was performed 6 h post-exposure. Biopsies were immunohistochemically stained, and lavage fluids analysed for soluble mediators.

**Results:**

In bronchial mucosal biopsies, nuclear translocation of the transcription factors aryl hydrocarbon receptor (AhR) and phosphorylated c-jun (p-c-jun) was significantly reduced within the bronchial epithelium after wood smoke exposure compared to filtered air. There was no endothelial adhesion molecule-mediated recruitment of neutrophils or other inflammatory cells into the bronchial mucosa.

**Conclusions:**

Exposure to wood smoke from incomplete wood log combustion suppressed nuclear translocation of transcription factors and the expected inflammatory response in endobronchial mucosal biopsies at 6 h post-exposure. This contrasts to the strong proinflammatory effects of other air pollutants such as ozone and diesel exhaust. Together with previous findings of increased cytotoxicity and impaired airway macrophage phagocytosis in humans, this response may be in line with compromised immune defence and increased susceptibility to airway infections, chronic bronchitis and COPD observed in populations exposed to high levels of indoor air pollution from wood smoke.

**Supplementary Information:**

The online version contains supplementary material available at 10.1186/s12989-026-00685-6.

## Background

Air pollution is linked to adverse health outcomes and is estimated to contribute to approximately 6.7 million premature annual deaths globally [1]. The Global Burden of Disease Study of 2021, presented in the Lancet, denotes particulate matter (PM) air pollution as the leading contributor to the global disease burden [2]. Particulate emissions from combustion of solid fuels are a major contributor to air pollution and more than 2.1 billion people are dependent on wood and other biomass fuels for their everyday life [1]. The use of simple stoves in confined spaces for cooking and heating in low and middle-income countries results in long-term indoor air exposures to biomass combustion-related PM at very high concentrations, i.e. 150–1000 μg/m^3^ and even higher [3, 4].

Wood smoke exposure has been shown to be a major contributor to the development and worsening of asthma, chronic bronchitis and chronic obstructive lung disease (COPD) [5–8]. It has also been highlighted that airway infections, including tuberculosis, increase with both chronic indoor and ambient exposures to wood smoke [9–11]. The reason why exposure to wood smoke is strongly associated with respiratory disease development and airway infections has not been properly clarified. Exposure to wood smoke and other biomass combustion emissions may be lower in more affluent regions but is still substantial across Europe and may in some locations contribute to at least 10–30% of the annual average of ambient fine PM [12]. Importantly, biomass burning is predicted to increase, both due to shortages of heating alternatives and European environmental policies [13, 14]. Increasing levels of ambient air pollution from incomplete combustion could therefore mean further challenges for respiratory and cardiovascular health [9, 15, 16].

The physicochemical properties and toxicity of wood smoke particles are heavily dependent on combustion conditions, temperature, air supply and fuel moisture [17, 18]. High combustion temperatures and optimized air supply, e.g. in modern pellet stoves, allow for more complete combustion, producing more alkali-rich ash particles. In contrast, incomplete combustion, e.g. air-starved and/or low temperature conditions, produces more organic PM and soot, which together with metal content have been associated with increased toxicity [19].

Controlled human exposure studies addressing health effects of biomass smoke have revealed inconsistent findings with no or only minor pro-inflammatory airway or systemic responses [20]. In a prior wood smoke exposure study, bronchoscopy was performed at 24 h post-exposure [21]. Contrary to our hypothesis, we did not observe a neutrophilic influx in bronchoalveolar lavage (BAL) or bronchial wash (BW), which is a classic hallmark of airway inflammation caused by air pollutants. Instead, indications of cytotoxicity were observed, characterized by reductions in macrophage, neutrophil and lymphocyte number in both BAL and BW. In bronchial mucosal biopsies, we demonstrated increased numbers of epithelial CD3+ and CD8+ lymphocytes as well as increased CD3+ lymphocytes and mast cells in the submucosa [21].

In a more recent study, we therefore investigated whether an earlier and transient airway inflammatory response in human subjects could be seen after a similar wood smoke exposure [22]. Bronchoscopy sampling was performed at 6 h after exposure. Lactate dehydrogenase (LDH) was increased 6 h after wood smoke exposure and no indications of neutrophilic influx in BAL or BW were noted, in similarity with the findings at 24 h post-exposure [21, 22]. Alveolar macrophages, which have a critical function by ingesting pathogens and modulating the immune defence, were found to be dysfunctional, with decreased phagocytic capacity. These indications of impaired cell function and cytotoxicity in airway samples from human subjects were also reflected in *in-vitro* cell cultures employing wood smoke PM generated from the same chamber exposure [22].

To follow up on these findings and further address the hypothesis that wood smoke, from a traditional heating stove, would cause an acute neutrophilic airway inflammation, we have in the present study conducted immunohistochemical analyses of endobronchial mucosal biopsies collected at 6 h after exposure to investigate early phase inflammatory responses within human bronchial wall tissue. Additionally, we hypothesized that wood smoke would activate transcription factors involved in redox-signalling and adhesion molecule expression, key components in the early phase of air pollutant-induced airway inflammation [23]. As a complement, BW and BAL were analysed for soluble markers of acute inflammation.

## Results

### Biopsies

Bronchial mucosal biopsies were sampled during bronchoscopy, from the proximal cristae of either the right or left bronchial tree 6 hours after exposure during a previous study [22].

**Fig. 1:**
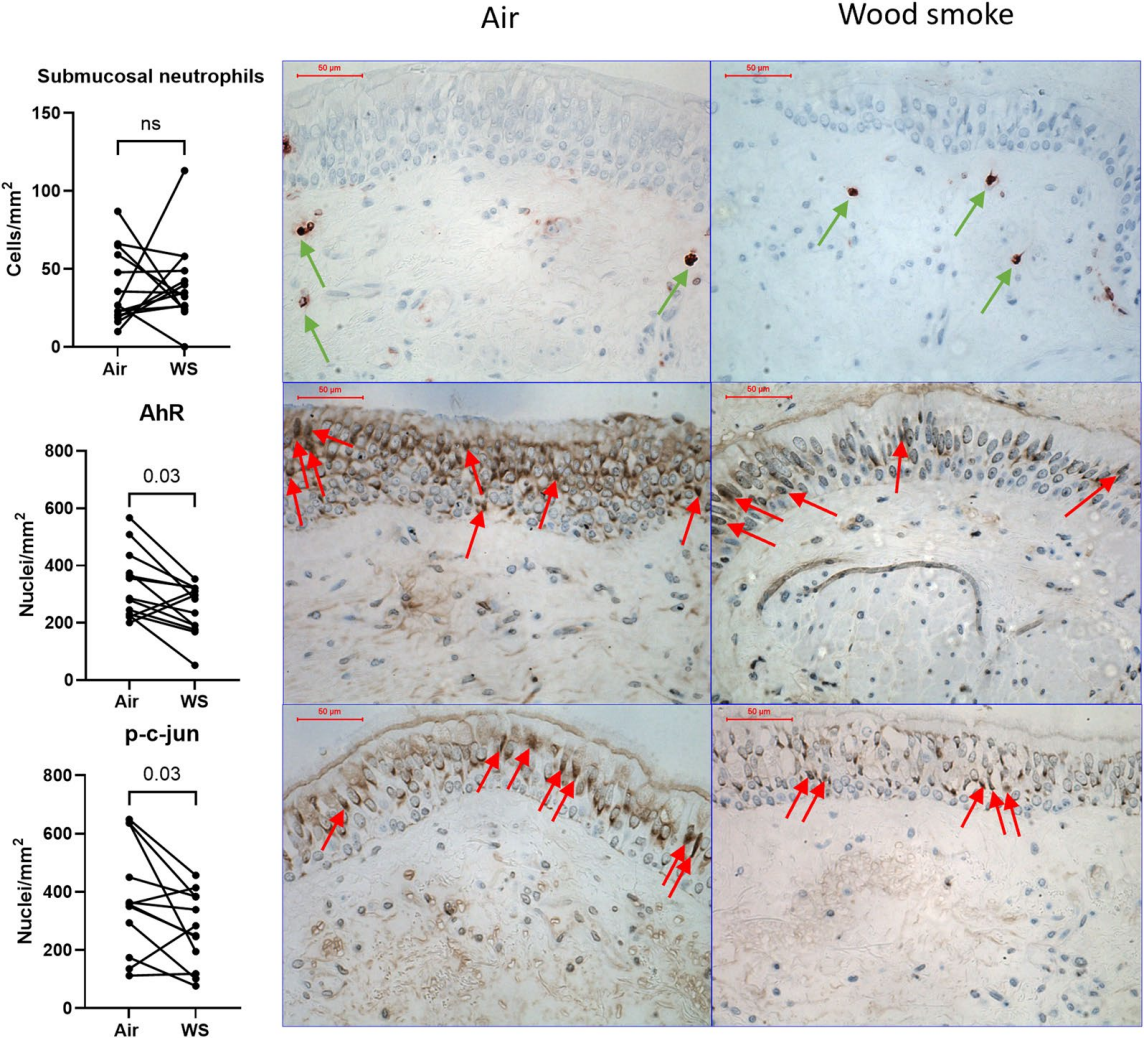
Immunohistochemical staining of bronchial biopsies. Immunohistochemical staining of bronchial mucosal biopsies sampled after filtered air and wood smoke (WS) exposures. All images with the same magnification (× 40). The red scale bar in the upper left corner indicates 50 µm. The first panel shows that there was no neutrophilic infiltration in the bronchial mucosa. Green arrows indicate positively stained neutrophils in the submucosa. Red arrows indicate positively stained nuclei. P-values were calculated using the Wilcoxon signed-rank test

Monoclonal antibodies were used to stain for Aryl hydrocarbon receptor (AhR), Aryl hydrocarbon receptor nuclear translocator (ARNT), Nuclear factor kappa B (NFκB), Nuclear factor erythroid 2-related factor 2 (Nrf2), NAD(P)H quinone dehydrogenase 1 (NQO1) and phosphorylated c-jun (p-c-jun). Of the stained panel of enzymes, nuclear translocation of p-c-jun (*p* = 0.03) and AhR (*p* = 0.03) decreased after wood smoke exposure, Table 1 and Fig. 1. Other transcription factors showed no clear tendency towards activation.

**Table 1:** Transcription factor and related enzymes expression in the bronchial epithelium

	Epithelium		
	Air	Wood smoke	*P*-value Air versus Wood smoke
AhR, total (%)	0.82 0.41–2.58	1.02 0.44–2.01	0.64
AhR, nuclei/mm^2^	286 227–404	283 183–322	0.03
ARNT, total (%)	0.2 0.02–0.7	0.2 0.07–1.0	0.89
ARNT, nuclei/mm^2^	216 125–407	208 98–287	0.41
NFκB, total (%)	1.1 0.5–2.2	1.7 0.7–3.6	0.64
NFκB, nuclei/mm^2^	153 101–363	209 92–297	0.64
Nrf2, total (%)	0.04 0.01–0.1	0.03 0.003–0.2	0.68
Nrf2, nuclei/mm^2^	22 0.0–41	0.0 0.0–68	0.94
NQO1, total (%)	2.2 1.3–4.5	3.1 2.2–5.5	0.37
NQO1, nuclei/mm^2^	–	–	–
p-c-jun, total (%)	3.1 1.6–4.5	2.2 1.8–3.6	0.09
p-c-jun, nuclei/mm^2^	357 204–590	267 138–384	0.03

Transcription factors and related enzymes in the bronchial epithelium after filtered air and wood smoke exposures. Data are presented as median with interquartile range. Total staining (cytoplasmic + nuclei) is expressed as % of the selected epithelial area. Staining of the nuclei is expressed as positively stained nuclei/mm^2^ of the selected epithelial area. Nuclear staining of NQO1 was not addressed, since it is primarily a cytosolic enzyme. P-values were calculated using the Wilcoxon signed-rank test

**Fig. 2:**
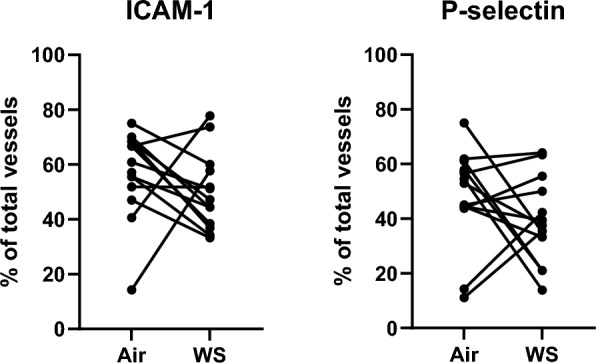
Expression of ICAM-1 and P-selectin on vascular endothelium. ICAM-1 and P-selectin positive vessels after air and wood smoke (WS) exposure. Total number of vessels was quantified through the pan-endothelial marker EN4. No significant difference was found between air and wood smoke exposure

The expression of the intercellular adhesion molecule-1 (ICAM-1) and P-selectin was quantified on the vascular endothelium through immunohistochemical staining. No significant difference in the expression of P-selectin or ICAM-1 was found in the vascular endothelium between the two exposures, see Fig. 2.

There were no significant changes in inflammatory cell counts in the bronchial mucosa or epithelium, see Table 2.

**Table 2:** Cell differential count in bronchial epithelium and submucosa

	Epithelium cells/mm			Submucosa cells/mm^2^		
			*P*-value			*P*-value
Cell marker	Air	Wood smoke	Air versus Wood smoke	Air	Wood smoke	Air versus Wood smoke
Neutrophils	1.0 0.0–1.4	1.2 0.0–2.6	0.58	27 20–61	35 26–51	0.90
CD68+ Macrophages	0.0 0.0–1.9	0.0 0.0–1.1	0.88	4.8 0.0–7.6	4.2 2.5–7.8	1.00
Eosinophils	0.0 0.0–0.0	0.0 0.0–0.0	0.50	9.9 4.6–19	5.8 3.5–20	0.33
Mast cells	0.0 0.0–2.0	1.0 0.0–2.5	0.15	30 20–44	32 22–41	0.80
CD3+ T-cells	3.8 1.4–16.0	6.3 1.7–11.3	0.91	93 54–181	131 72–207	0.58
CD4+ T-helper cells	1.2 0.0–2.5	0.86 0.0–4.8	0.81	45 24–79	67 41–88	0.19
CD8+ T-cytotoxic cells	3.6 1.5–7.3	3.5 1.7–6.8	0.41	38 28–94	52 39–81	0.46
CD56+ NK-cells	0.0 0.0–1.8	0.72 0.0–1.3	0.92	2.7 0.0–5.0	2.9 1.1–5.8	0.46

Inflammatory cell counts in the bronchial epithelium and submucosa of immunohistochemically stained endobronchial mucosal biopsies. Data are expressed as cells/mm of epithelial length and cells/mm^2^ of submucosa and presented as median with interquartile range

### BW and BAL

Wood smoke caused only a small, albeit significant, increase in Interleukin 6 (IL-6) in BW (p = 0.035), as shown in Table 3. Exposure to wood smoke did not cause any significant change in the cytotoxic T-cell marker Granzyme A (GrzA), the neutrophil proteases; matrix metalloproteinase 9 (MMP-9) and myeloperoxidase (MPO) or the tissue inhibitor of metalloproteinase (TIMP1), compared with air exposure.

**Table 3:** Soluble inflammatory markers in BW and BAL

	BW			BAL		
			*P*-value			*P*-value
Inflammatory marker	Air	Wood smoke	Air versus Wood smoke	Air	Wood smoke	Air versus Wood smoke
IL-6 pg/ml	5.8 3.1–8.4	8.5 3.1–22	0.035	0.9 0.8–1.5	1.3 0.7–1.9	0.051
GrzA pg/ml	68 7.8–134	49 15–147	0.76	8.3 1.8–20	3.0 0.2–40	0.73
MPO ng/ml	19 13–25	16 12–26	0.77	2.9 1.9–4.4	2.9 1.8–5.3	0.27
MMP-9 ng/ml	2.8 1.3–4.7	3.7 1.3–7.0	0.33	0.2 0.1–0.5	0.2 0.2–1.1	0.22
TIMP1 ng/ml	5.9 4.2–8.2	4.8 3.3–6.3	0.22	0.8 0.4–1.1	0.8 0.5–1.6	0.36

Soluble inflammatory markers in BAL and BW 6 h after exposure to filtered air and wood smoke. Data are expressed as median with interquartile range

## Discussion

Contrary to our hypothesis, exposure to wood smoke in human subjects did not induce bronchial mucosal neutrophilia or influx of other inflammatory cell types at the investigated time point, 6 h after exposure, complementary to our preceding investigation conducted 24 h following a similar wood smoke exposure [21]. Neither was the expression of vascular adhesion molecules, key factors for cell migration, upregulated at this time point. Of note, nuclear translocation of the transcription factors p-c-jun and AhR was suppressed following exposure to wood smoke, and a limited pro-inflammatory response was observed in terms of a moderate increase in IL-6 in BW.

Neutrophilic inflammation may result in tissue damage, but may also, together with alveolar macrophages, play an important role in clearing damaged and dead cells. This contrasts to findings from exposure studies employing diesel exhaust or ozone. These pollutants induce a pronounced influx of neutrophils, lymphocytes, macrophages and mast cells into the bronchial mucosa and bronchoalveolar lavage fluids, with upregulated transcription factors, kinases, cytokine release and vascular adhesion molecule expression [24–29]. However, the studied type of wood smoke cannot be considered harmless, as we have shown both impaired alveolar macrophage phagocytic capacity ex vivo [22] and reduced airway inflammatory cell numbers in humans 24 h post-exposure [21]. Overall, these data indicate that this type of wood smoke exposure induces cytotoxicity and immune cell dysfunction, without an inflammatory response.

Increased expression of vascular adhesion molecules in the bronchial submucosa is, together with cytokine release, central for initiating and enabling cell recruitment from the blood stream into the bronchial tissue. P-selectin is important for the early phase of adhesion-rolling of leukocytes along the vascular endothelium, and ICAM-1 for the firm adhesion and migration of cells into the bronchial wall [30]. Neither was found to be upregulated at 6 h after wood smoke exposure. P-selectin and ICAM-1 have previously been shown to be upregulated in endobronchial mucosal biopsies after ozone and diesel exhaust exposures in humans, at 1, 5 and 6 h, respectively [24, 31].

Exposure to wood smoke has previously been associated with oxidative stress and inflammation [7, 32]. Air pollution particles that deposit in the airways and encounter alveolar macrophages, bronchial epithelial cells and pneumocytes can cause reactive oxygen species production in these cell types and, thus, oxidative stress [32–34]. Oxidative stress can promote inflammation through several different pathways [35]. Redox-sensitive transcription factors may increase vascular adhesion molecule expressions, together with chemokine and cytokine production. Activation of transcription factors, such as Nrf2, AhR and NFκB, has been observed in controlled human exposure studies to other air pollutants [34, 36–38]. P-c-Jun forms, together with c-Fos, the transcription factor Activator-protein-1 (AP-1), an early-response transcription factor that regulates genes involved in cellular antioxidant defense mechanisms [39, 40]. We found, p-c-jun expression to be decreased following wood smoke exposure, contrary to our original hypothesis. *In-vitro* findings of increased nuclear p–c-jun 4–8 h after a heat shock support an appropriate timing to address p-c-jun response [40].

Aryl hydrocarbon receptor (AhR) is a transcription factor and sensor for foreign xenobiotic compounds such as polycyclic aromatic hydrocarbons (PAH) and quinones from combustion. They can bind to AhR in the cytoplasm leading to translocation to the nucleus and binding to ARNT [41]. This promotes the transcription of cytochrome *P*-450 1A1 (CYP1A1), Nrf2 and NQO1, key players in phase I detoxification and the immune response [42]. When employing the same techniques as in the present study, it was shown that PAH-containing air pollutants, such as diesel exhaust, are normally strong activators of AhR in human airways, accompanied by inflammation [38, 43, 44]. The investigated wood smoke was generated from incomplete combustion and rich in PAHs [22], but a suppressed AhR response was found, with no change in Nrf2 and NQO1 expression. However, AhR signaling dynamics have been shown to be rapid and transient, as recently described *in-vitro* in a human epithelial cell line exposed to wood smoke particles [45]. It can therefore not be excluded that an early activation of ARNT and AhR, before the 6 h bronchoscopy, might have suppressed transcription factors such as NFκB and others, as well as subsequent mediator production. On the other hand, an earlier transient AhR response should have upregulated Nrf2 and NQO1 and led to an inflammatory response. We have previously found early suppression of key transcription factors in bronchial biopsies from human subjects exposed to the oxidative air pollutant ozone, but this was followed by an extensive neutrophil-dominated inflammatory response [46].

When considering soluble components, the only indicator of an active inflammation was a mild increase in IL-6 in BW, but no other inflammatory marker was increased; GrzA, MMP-9, MPO in BW or BAL, and there was no acute response identified in terms of a neutrophilic influx in the bronchial mucosa at 6 h, or as previously shown at 24 h [21, 22].

Taken together, through suppression of key transcription factors, wood smoke exposure could potentially result in less effective xenobiotic detoxification processes, that are necessary for tissue protection and avoidance of cellular dysfunction. We have previously seen indications of such effects in human airways at the 24-h bronchoscopy sampling timepoint [21]. Increased LDH levels, loss of bronchial cells as well as impaired macrophage function and phagocytosis at 6 h post exposure [22]. Wood smoke exposure has indeed been strongly associated with an increase in airway symptoms and respiratory infections [10, 47, 48]. Furthermore, on a global scale, wood smoke exposure is a major risk factor for the development of chronic bronchitis and COPD, with COPD being the third leading cause of death worldwide [5, 8].

### Strengths

The study was part of a pre-planned sequence of controlled and well-characterized wood smoke exposure investigations in humans, designed to provide detailed data on airway responses to wood smoke. Investigating bronchial mucosal biopsies offers a unique opportunity to study detailed processes to environmental challenges in the bronchial epithelium, in this study design with a real-world exposure scenario. All exposures to filtered air and wood smoke were performed in a randomised, blinded and cross-over fashion. This design provides high internal validity, as each participant in the study serves as his/her own control. Direct comparisons can be done to a sequence of previous studies in human subjects, using similar exposure protocols and sampling techniques, with air pollutants such as nitrogen dioxide, ozone, diesel exhaust, organic dust and endotoxin. Relevant exposure doses were chosen to reflect real-life exposure scenarios in Europe as well as in developing countries. Detailed physicochemical analyses by these investigators have ensured relevance for the wood smoke exposures and chemical composition of the PM [22].

### Limitations

Not all exposures to wood smoke and other types of biomass burning emissions are similar, since exhaust characteristics from biomass burning vary heavily depending on combustion conditions [17, 18]. This limits the generalisation of the present findings. This study was aimed to explore the health effects of wood smoke exposure from rather inefficient and poor combustion in a traditional heating stove, with high soot and PAH levels, which is a common exposure situation in many parts of the world [49]. When comparing studies, it is important to recognize that wood and biomass smoke exposure characteristics can be more heterogenous than exposure to e.g. diesel engine exhaust [50]. This concerns both *in-vitro* and human exposure studies. Due to the invasive nature of the bronchoscopy procedure, we have been limited to one time point post-exposure and have not been able to perform repeated endobronchial mucosal sampling at several time points in the same individual. Instead, each time point for bronchoscopy sampling for studies of time kinetics have demanded separate studies. The exposure setup is limited to a single exposure and does not necessarily reflect chronic exposure scenarios. For this study, healthy young adults were recruited, who are likely more resilient to the effects of air pollution than older individuals with pre-existing medical conditions. Despite these limitations, the controlled exposure setup remains an important model for real-life exposure to wood smoke.

## Conclusions

Controlled chamber exposure in humans to wood smoke from incomplete combustion suppresses the expression of the key transcription factors AhR and p-c-jun, in bronchial epithelial cells at 6 h after exposure. This could potentially lead to less effective xenobiotic detoxification processes, tissue damage and cellular dysfunction. Such effects were indeed identified in the preceding study, which showed no clear inflammatory response at 6 h in BW and BAL, but instead increased LDH levels and impaired macrophage phagocytosis. These findings contrast to the airway responses seen after exposure to other air pollutants. Cytotoxicity and cellular dysfunction may contribute to the extensive negative health effects associated with biomass smoke exposure globally, in terms of chronic bronchitis, COPD and pneumonias.

## Methods

### Study design

Information regarding participants, study design and exposure has been described previously [22]. Briefly, fourteen, non-smoking, healthy participants (mean age 26, range 19–35 years, 3 females, 11 males) were recruited. Exposures were performed in a double-blind, cross-over fashion, where all participants were exposed to both wood smoke and filtered air, at least 3 weeks apart. Wood smoke was generated through incomplete wood log combustion in a common residential Swedish wood stove and was diluted to achieve a fine particulate matter < 1 µm (PM_1_) concentration of ∼450 µg/m^3^. Exposures lasted for 2 h. The mean PM_1_ concentration in the chamber was 409 ± 43 µg/m^3^ (filter based). A detailed report on exposure characteristics has been previously published [22].

### Bronchoscopy

Bronchoscopy was performed 6 h after exposure using a flexible bronchoscope (EB-580S, FUJIFILM Corporation, Japan). Before the procedure, topical anesthesia with Lidocaine was applied to the pharynx, epipharynx and bronchial tree. During bronchoscopy, endobronchial mucosal biopsies were sampled from proximal cristae of either the right or left bronchial tree, with BW (2 × 20 ml) and BAL (3 × 60 ml) collection performed with sterile saline on the contralateral side. Aspirates from BAL and BW were placed in separate siliconized containers, kept on ice and later filtered through a nylon filter (100 µm). Mucosal biopsies were first placed in ice-cold acetone, containing protease inhibitors overnight and then processed and embedded in glycol methacrylate (GMA) resin (Polyscience; Northampton, England). GMA blocks were stored in airtight containers at -20 degrees Celsius until sectioning and immunohistochemical analysis.

### BAL/BW

The lavage samples were centrifuged at 400 × g for 15 min. Supernatants were aliquoted and frozen at − 80 °C and later analyzed for IL-6, GrzA, MMP-9, MPO and TIMP1, (R&D Systems, Abingdon, UK), according to the manufacturer’s instructions.

### Immunohistochemistry

Bronchial mucosal biopsies were cut into 2 µm thick sections, placed floating in ammonia water (1:500), picked up onto poly-L-lysine glass slides and then dried at room temperature for at least an hour. Primary antibodies were used to stain the following markers: vascular endothelial adhesion molecules, inflammatory cells and transcription factors, see Additional file 1 for the complete list of antibodies used and their respective suppliers. Slides were first treated with 0.1% sodium azide and 0.3% hydrogen peroxide in distilled water to block endogenous peroxidases. Sections were then rinsed with TRIS-buffered saline (TBS). To block nonspecific antibody binding undiluted culture medium (DMEM, Sigma) containing 10% fetal calf serum and 1% bovine serum albumin was applied for 30 min. Slides were then drained and monoclonal antibodies were applied and incubated at room temperature overnight. After rinsing with TBS, a secondary antibody against the primary antibody was added for 2 h and slides were once again rinsed with TBS. For vessels and cell markers (EN4, P-selectin, ICAM-1, NE, Eos, MC, CD3+, CD4+, CD8+, CD56+ and CD68+), 3-aminoethyl carbazole (AEC) (Vector laboratories, Burlingame, USA) was added and incubated for 15 min to develop a red colour. Slides were then rinsed with TBS. All sections were counterstained with Mayer’s hematoxylin. For negative controls, the primary antibody was omitted in the sections.

Positively stained cells and vessels were counted in airway epithelium and submucosa respectively, excluding submucosal glands, blood vessels, and smooth muscle. Counts were adjusted for submucosal area and epithelial length using LeicaQWin V3 (Leica Q500IW, Leica, Cambridge, United Kingdom). Activated blood vessels stained with p-selectin and ICAM-1 were counted and then expressed as a percentage of the total number of vessels stained with the pan-endothelial marker EN4.

When it comes to staining procedures for transcription factors, supplementary steps were taken to increase the permeability of cells. TBS with 0.3% Triton X-100 was applied for 30 min, before the staining procedure described above. Sections were then visualized with 3,3-diaminobenzidine (DAB) to develop a brown color. Transcription factors and enzymes in the bronchial epithelium were quantified using a Leica DFC 320 camera (Leica, Cambridge, United Kingdom) connected to a workstation with LeicaQWin V3. Detection of an appropriate colour was quantified using binary definition of colour. The binary image required the user to define which pixel in the image to be considered for measurement and was performed as described previously [36]. Positive pixels were then adjusted with the measured epithelial area and expressed as percentage of total staining area (both cytoplasmic and nuclear staining). It was possible to distinguish between nuclear and cytoplasmic staining using a light microscope. Positively stained nuclei in the epithelium were counted and expressed as positive nuclei/mm^2^. All quantification of the immunohistochemical staining was performed by one person unaware of the coding. All analyses were performed double-blinded. Codes were broken after statistical analysis was completed.

### Statistics

All data were tested for normality with the Shapiro–Wilk test. Non-parametric statistical analyses were performed for comparison of BW, BAL and biopsy data. Wilcoxon signed-rank test was used for the paired biopsy, BW and BAL analyses. Values of *p* < 0.05 were considered significant. Statistical analyses were performed with Prism 10 (Graph Pad software for Windows, San Diego, USA) and SPSS, version 26 for Windows (IBM® SPSS® Statistics 20, Chicago, USA).

## Supplementary Information

Additional file1.

## Data Availability

All relevant data are included in the manuscript and supporting information. They are also available from the authors upon request.
