# Supplementary material for: Bronchial mucosal nuclear transcription factor expression and inflammatory response in humans after exposure to wood smoke
Source: Part Fibre Toxicol. 2026 Jun 12;23:32. doi: 10.1186/s12989-026-00685-6 (PMC13270798; doi:10.1186/s12989-026-00685-6)
Supplement: Supplementary file 1 — Additional file1. [file 12989_2026_685_MOESM1_ESM.docx]

**Additional file 1.**

**Table. Antibodies used for immunohistochemical staining.**

| **Antibody** | **Marker for** | **Company** |
| --- | --- | --- |
| CD3 | CD3 (T-Lymphocyte) | Biolegend, San Diego, USA |
| CD4 | CD4 (T helper cell) | Biolegend, San Diego, USA |
| CD8 | CD8 (cytotoxic T-cell) | Agilent Dako, Santa Clara, USA |
| CD56 | Natural killer cell (NK-cell) | Agilent Dako, Santa Clara, USA |
| CD68 | Macrophage | Agilent Dako, Santa Clara, USA |
| NE | Neutrophil elastase | Agilent Dako, Santa Clara, USA |
| MC | Mast cell elastase | Agilent Dako, Santa Clara, USA |
| Eos | Eosinophil cation protein | Diagnostics development, Uppsala, Sweden |
| P-sel | P-selectin (CD62p). Adhesion molecule | Bio-Rad, Hercules, USA |
| ICAM-1 | Intercellular Adhesion Molecule 1, CD54. Adhesion molecule. | Invitrogen, Carlsbad, USA |
| EN4 | Endothelial pan antibody | MONOSAN Sanbio, Uden, Netherland |
| AHR | Aryl hydrocarbon receptor | Santa Cruz, Dallas, USA |
| ARNT | Aryl hydrocarbon receptor nuclear translocator | Santa Cruz, Dallas, USA |
| p-c-jun | Phosphorylated c-jun | Santa Cruz, Dallas, USA |
| NQO1 | NAD(P)H quinone dehydrogenase 1 | Santa Cruz, Dallas, USA |
| Nrf2 | Nuclear factor erythroid 2-related factor 2 | Santa Cruz, Dallas, USA |
| P65 | P65 (NF-κB subunit) | BD Pharmingen, Becton, USA |
| Secondary antibody | Biotinylated rabbit anti mouse | Agilent Dako, Santa Clara, USA |
